# B7 Family Members in Lymphoma: Promising Novel Targets for Tumor Immunotherapy?

**DOI:** 10.3389/fonc.2021.647526

**Published:** 2021-03-31

**Authors:** Wei Zhang, Yu Qiu, Xiaoli Xie, Yao Fu, Lijuan Wang, Zhen Cai

**Affiliations:** ^1^ School of Clinical Medicine, Binzhou Medical University, Yantai, China; ^2^ Central Laboratory, Linyi People’s Hospital, Linyi, China; ^3^ Bone Marrow Transplantation Center, Department of Hematology, The First Affiliated Hospital, College of Medicine, Zhejiang University, Hangzhou, China

**Keywords:** lymphoma, PD-L1, PD-L2, B7-H2, B7-H3, B7-H4, B7-H6

## Abstract

T cells play a vital role in the immune responses against tumors. Costimulatory or coinhibitory molecules regulate T cell activation. Immune checkpoint inhibitors, such as programmed cell death protein 1 (PD-1) and programmed death ligand 1 (PD-L1) have shown remarkable benefits in patients with various tumor, but few patients have displayed significant immune responses against tumors after PD-1/PD-L1 immunotherapy and many have been completely unresponsive. Thus, researchers must explore novel immune checkpoints that trigger durable antitumor responses and improve clinical outcomes. In this regard, other B7 family checkpoint molecules have been identified, namely PD-L2, B7-H2, B7-H3, B7-H4 and B7-H6. The aim of the present article was to address the expression, clinical significance and roles of B7 family molecules in lymphoma, as well as in T and NK cell-mediated tumor immunity. B7 family checkpoints may offer novel and immunotherapeutic strategies for patients with lymphoma.

## Introduction

T cells play important roles in antitumor immunity, and their dysfunction results in immune evasion (1). It is unclear how tumor interact with the immune system. Immunotherapeutics that target checkpoints have achieved remarkable clinical responses in tumor treatment. However, many patients remain unresponsive to such therapies, suggesting that there are other mechanisms of T cell exhaustion (2). Thus, researchers must investigate novel co-inhibitory molecules for immunotherapy. Combining new target molecules with present immunotherapies may offer novel strategies and improve clinical responses.

**Figure 1:**
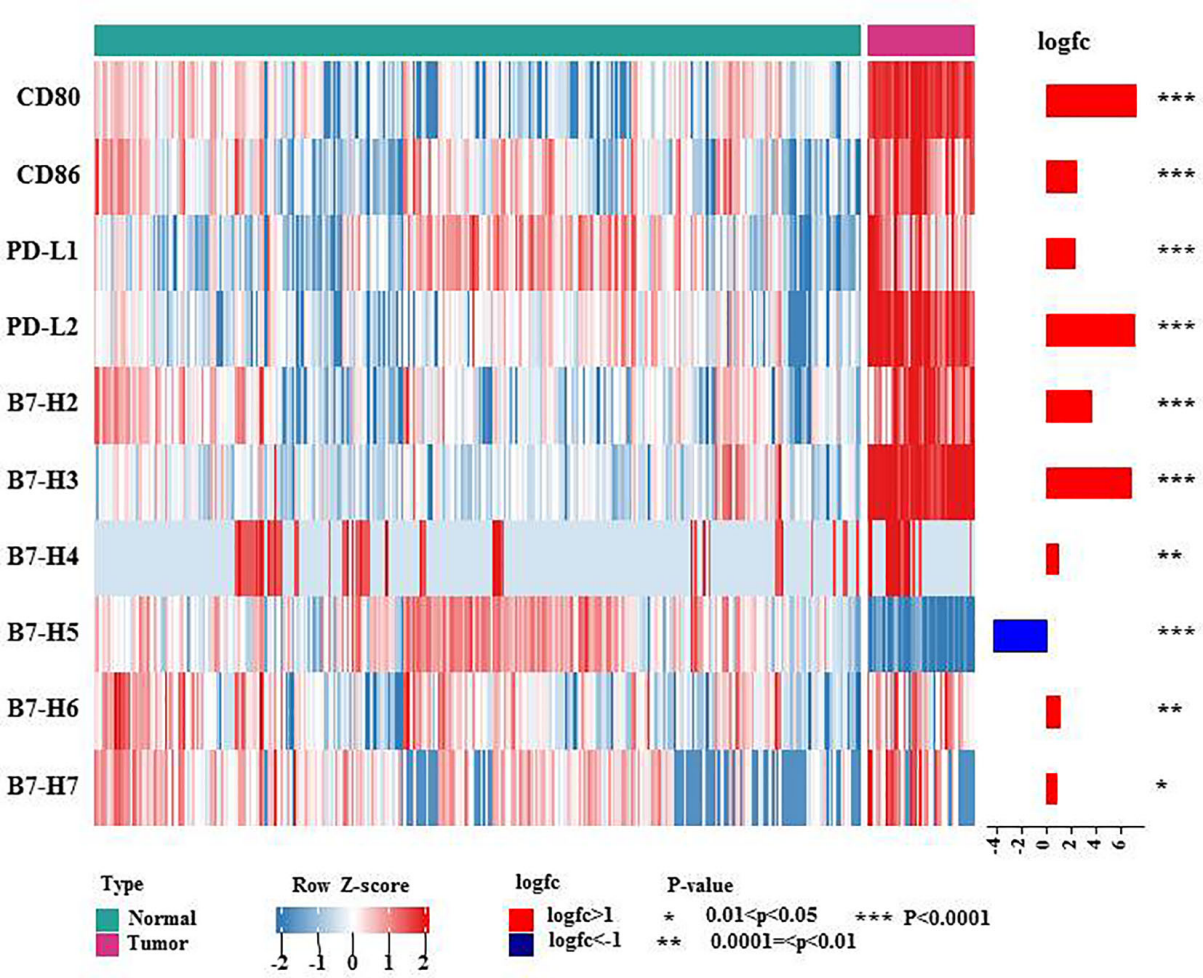
mRNA expression levels of B7 family molecules in DLBCL. This heatmap shows the expression of the 10 genes of B7 family members in normal tissue and DLBCL: normal tissue from the whole blood cells of GTEx (n = 337), DLBCL from TCGA DLBCL (n= 47). The data were obtained from UCSC Xena and were log2 transformed and were analyzed by Mann-Whitney U test. * 0.01<*P*<0.05, ** 0.0001=<*P*<0.01, *** *P*<0.0001.

B7 family members have received attention because they are expressed on T cells in cases of immune evasion and tumorigenesis. To date, ten B7 family molecules have been identified: B7-1 (CD80), B7-2 (CD86), B7-H1 (PD-L1, CD274), B7-DC (CD273 or PD-L2), B7-H2 (ICOSLG, CD275), B7-H3 (CD276), B7-H4 (B7S1, B7x or VTCN1), B7-H5 (VISTA, GI24, or PD-1H), B7-H6 (NCR3LG1) and B7-H7 (HHLA2) (3). Using the TCGA and GTEx databases, we investigated the mRNA expression levels of B7 family proteins in lymphoma. As shown in the heatmap showed (**Figure 1**), all B7 family members reported in the literature other than B7-H5 were more highly expressed in diffuse large B cell lymphoma (DLBCL), suggesting that these molecules may play vital roles in lymphoma immunity, explaining the poor effect of PD-1/PD-L1 therapy.

The expression of B7 family molecules were regulated by various mechanisms and play important roles in lymphoma proliferation, migration, evasion, chemoresistance and immune evasion. Blockade of B7 family molecules released T/NK cells from the inhibitory effects and restores antitumor immunity *via* promoting T/NK cell activation, proliferation and cytotoxicity, and suppressing inhibitory immune cells and molecules. Immunotherapies targeting B7 family members include monoclonal antibody (mAb), inhibitors, antibody-drug conjugates (ADCs), single chain fragment variables (scFvs), antibody-dependent cell-mediated cytotoxicity (ADCC), bispecific T cell engagers (BiTEs) and chimeric antigen receptor (CAR) T cell therapy (4).

The present review summarizes the research involving B7 family members in lymphoma, namely PD-L1, PD-L2, B7-H2, B7-H3, B7-H4 and B7-H6. The surface expression of these molecules in lymphoma is shown in in **Figure 3**. Further exploration of these molecules is needed to develop effective immunotherapies, either as monotherapy or in combination with other antibodies.

## 2 PD-L1

The PD-1/PD-L1 axis is a vital checkpoint in tumor progression and immune evasion. The binding of PD-1 to PD-L1 resulted in T cell anergy, exhaustion, apoptosis, and reduced cytotoxicity (5). The drug mechanisms of anti- PD-1/PD-L1 antibodies are similar. They destroy the immunosuppressive microenvironment and reactivate T cells, allowing them to recognize and kill tumor cells by blocking the binding of PD-L1 on tumor cells to PD-1 on T cells (6).

Anti-PD-1 antibodies have been approved for use in various solid tumors and lymphomas (7). A multicenter, single-arm, phase II trial of sintilimab to treat relapsed or refractory classical Hodgkin’s lymphoma (cHL) was carried out in China and showed that the overall response rate (ORR) was 80.4% (5, 8). Single sintilimab therapy also revealed an anti-tumor effect in extranodal natural killer (NK)/T cell lymphoma (ENKTL) in a phase II trial (5). The ORR was 67.9% and the disease control rate was 85.7% (5). Sintilimab combined with decitabine and the histone deacetylase inhibitor chidamide resulted in partial remission in DLBCL (9). Phase II studies have revealed that the ORR after treatment with either nivolumab or pembrolizumab was 66.3% and 69.0%, respectively, in patients with relapsed or refractory cHL (5, 10, 11). The ORR of pembrolizumab in patients with ENKTL was 78.6% (12). A recent study reported that geptanolimab showed promising efficacy and manageable toxicity in patients with relapsed/refractory peripheral T cell lymphoma (13). Interestingly, patients with PD-L1 expression > 50% obtained more benefit from geptanolimab treatment, with an ORR of 53.3% and a median progression-free survival of 6.2 months (13). The correlations between PD-L1 expression and response to anti-PD-1 antibodies should to be further investigated in future clinical trials. We have summarized the finished clinical trials in **Table 1** and ongoing ones in **Table 2**.

**Table 1:** The finished clinical trials targeting on B7 family molecules in lymphoma.

Targets	Drug	Trial ID	Phase	N	Diagnosis	Response	Ref
PD-L1	Avelumab	NCT03439501	II	21	ENKTCL	CR 24%, ORR 38%	(14)
PD-L1	Durvalumab	NCT02401048	Ib/2	61	r/r DLBCL, r/r FL	ORR 25%	(15)
PD-L1	Atezolizumab	NCT02541604	I/II	90	r/r solid tumors and lymphoma	ORR 5% SD 13%	(16)
PD-1	CD19-PD-1/CD28-CAR T cell	NCT03258047	Ib	17	PD-L1+ LBL	CR 41.2%, ORR 58.8%	(17)
PD-1	Nivolumab	NCT01592370	II	23	r/r HL	ORR 87%, CR 17%, PFS rate 86%	(18)
PD-1	Nivolumab	NCT02181738	II	80	cHL	ORR 66.3%, CR 8.8%, PR 57.5%, PFS 10 m	(10)
PD-1	Nivolumab Ibrutinib	NCT02329847	I/II	144	DLBCL, FL	CR 61%, PR 14%, SD 3%	(19)
PD-1	Pembrolizumab	NCT01953692	Ib	31	r/r cHL	ORR 65%, CR 16%,	(20)
PD-1	Pembrolizumab	NCT01953692	Ib	21	r/r PMBL,	ORR 48%, CR 33%	(21)
PD-1	Pembrolizumab	NCT02576990	II	53	r/r PMBL,	ORR 45%, CR 13%	(21)
PD-1	Pembrolizumab	NCT02332980	II	9	r/r DLBCL	ORR 44%	(22)
PD-1	Pembrolizumab	NCT02453594	II	210	r/r cHL	ORR 71.9%, CR 27.6%, PR 44.3%, PFS 13.7 m, DOR 16.6 m, 3 years OS 86.4%	(23)
PD-1	Pembrolizumab R-CHOP	NCT02541565	1	33	DLBCL, FL	ORR 90%, CR 77%, PFS 83%	(24)
PD-1	Pembrolizumab+ Vorinostat	NCT03150329	I	30	DLBCL, PMBL, FL, cHL	ORR 30%, CR 30%, DOR 6 m, PFS 59%	(25)
PD-1	Camrelizumab	NCT03155425	II	75	cHL	CR 28%, PR 48%	(26)
PD-1	Tislelizumab	NCT03209973	II	70	r/r cHL	PR 87.6%, CR 62.9%, ORR 87.1%, CR 62.9%, 9 m PFS=74.5%.	(27)
PD-1	Nivolumab Brentuximab Vedotin	NCT02581631	I, II	30	PMBL	ORR 73%, CR 37%	(28)
PD-1	Ipilimumab Nivolumab	NCT01822509	I	28	hematologic cancer	ORR 32%, PFS 1 year	(29)
PD-1	Geptanolimab	NCT03502629	II	102	r/r PTCL	OR 40.4%, CR 14.6%, PR 25.8%, DOR 11.4 m	(13)

cHL, classical Hodgkin Lymphoma; DLBCL, Diffuse Large B-Cell Lymphoma; ENKTCL, Extranodal Natural Killer/T-cell Lymphoma; FL, Follicular Lymphoma; LBL, large B-cell lymphoma; m, months; N, number; PMBL, primary mediastinal lymphoma; PTCL, Peripheral T-cell Lymphoma; r/r, relapsed or refractory; PMBL, Primary Mediastinal Large B-cell Lymphoma; CR, complete response; DOR, Duration of overall response; ORR, overall response rate; OS, overall survival; PFS, progression-free survival; PR, partial response; SD, stable disease.

**Table 2:** The ongoing clinical trials targeting on B7 family molecules.

Targets	Drug	Disease	Phase	Status	Trial ID
PD-L1	Durvalumab lenalidomide	NKTCL	II	Not yet recruiting	NCT03054532
PD-L1	Durvalumab Rituximab Acalabrutinib	PCNSL	I	Not yet recruiting	NCT04688151
PD-L1	Acalabrutinib Durvalumab	PCNSL SCNSL	I	Not yet recruiting	NCT04462328
PD-L1	Atezolizumab	DLBCL		Recruiting	NCT03850028
PD-L1	Atezolizumab Rituximab Gemcitabine Oxaliplatin	r/r DLBCL	II	Active, not recruiting	NCT03422523
PD-L1	Atezolizumab	CTCL,SS	II	Active, not recruiting	NCT03357224
PD-L1	Avelumab	r/r ENKTCL	II	Active, not recruiting	NCT03439501
PD-L1	Avelumab	Advanced HL	II	Recruiting	NCT03617666
PD-L1	Avelumab	PTCL	II	Active, not recruiting	NCT03046953
B7-H3	B7-H3 CAR T	DIPG, DMG, r/r CNS tumors	I	Recruiting	NCT04185038
B7-H3	B7-H3 CAR T	r/r Glioblastoma	II	Recruiting	NCT04077866
B7-H3	B7-H3 CAR T	r/r solid tumors	I	Recruiting	NCT04483778
B7-H3	4SCAR-276	solid tumors	I/II	Recruiting	NCT04432649
B7-H3	B7-H3 CAR T, Fludarabine, Cyclophosphamide	Epithelial Ovarian Cancer	I	Not recruiting	NCT04670068
B7-H3	MGA271	Prostate Cancer	II	Active, not recruiting	NCT02923180
B7-H3	Enoblituzumab, Retifanlimab, Tebotelimab	Head and neck caner	II	Not recruiting	NCT04634825
B7-H3	MGC018	Advanced solid tumors	I	Recruiting	NCT03729596
B7-H3	MGD009	Advanced solid tumors	I	Active, not recruiting	NCT02923180
B7-H4	FPA150	Advanced solid tumors	I	Active, not recruiting	NCT03406949
B7-H6	BI 765049 BI 754091	Advanced solid tumors	I	Not recruiting	NCT04752215

CNS, central nervous system; CTCL, cutaneous T‐cell lymphoma; DIPG, diffuse intrinsic pontine glioma; DMG, diffuse midline glioma; DLBCL, diffuse large B-cell lymphoma; ENKTCL, extranodal natural killer/T-cell lymphoma; HL, Hodgkin Lymphoma; PCNSL, primary central nervous system lymphoma; PTCL, Peripheral T-cell Lymphoma; r/r, relapsed or refractory; SCNSL, secondary central nervous system lymphoma; SS, Sezary syndrome.

The efficacy and safety of anti-PD-L1 antibodies in lymphoma patients have also been assessed in clinical trials, involving patients with lymphoma. At present, the following anti-PD-L1 antibodies are used in clinical practice: avelumab, durvalumab and atezolizumab. The finished clinical trials are summarized in **Table 1** and ongoing ones in **Table 2**. Avelumab is a fully human IgG1 mAb that selectively blocks PD-L1 and enhances anti-tumor T-cell activity (7). A phase I study of avelumab demonstrated that ORR and complete response (CR) were 54.8% and 6.5%, respectively, in patients with relapse/refractory cHL who had suffered progression following stem cell transplantation (SCT) or SCT-ineligible (7). A phase II trial demonstrated that the CR of avelumab was 24% and that the ORR was 38% in patients with relapsed or refractory ENKTL (14). The response to avelumab was strongly correlated with PD-L1 expression in tumor tissues (14). A phase I b/2, multicenter, open-label study of ibrutinib plus durvalumab in relapsed/refractory follicular lymphoma (FL) or DLBCL showed ORR values of 25% among all patients, 26% among patients with FL, and 13% among patients with germinal center B-cell DLBCL (15). A multicenter open-label, phase I-II trial of patients with solid tumors or lymphomas observed that atezolizumab was well tolerated with generally comparable exposure across populations (16). A phase Ib study involving patients with PD-L1+ large B-cell lymphoma demonstrated that the ORR of CD19-PD-1/CD28-CAR T-cell therapy was 58.8%, and that the CR was 41.2%. No severe cytokine release syndrome or neurologic toxicity was reported in that study (17).

One recent study reported that high levels of plasma-soluble PD-L1 and signal transducer and activator of transcription (STAT) 3 were related to worse progression-free survival and overall survival in patients with DLBCL (30). Another study showed that vincristine induced PD-L1 expression *via* p-STAT3 and augmented the efficacy of PD-L1 blockade therapy by activating effector T cells and increasing the antitumor immune response in DLBCL (31). The expression levels of PD-L1 on monocytes are increased in patients with NK/T-cell lymphoma and constitute a novel predictor of prognosis (32). More clinical trials involving anti-PD-1/PD-L1 antibodies are currently ongoing in patients with lymphoma.

## 3 PD-L2

Programmed death ligand-2 (PD-L2), is a PD-1 receptor. It is mainly expressed in dendritic cells, macrophages, mast cells and B cells, as well as in hematological malignancies, including multiple myeloma (MM), acute leukemia and chronic lymphocytic leukemia (33). However, it has little or no significant effect on prognosis in these diseases (33).

**Figure 2:**
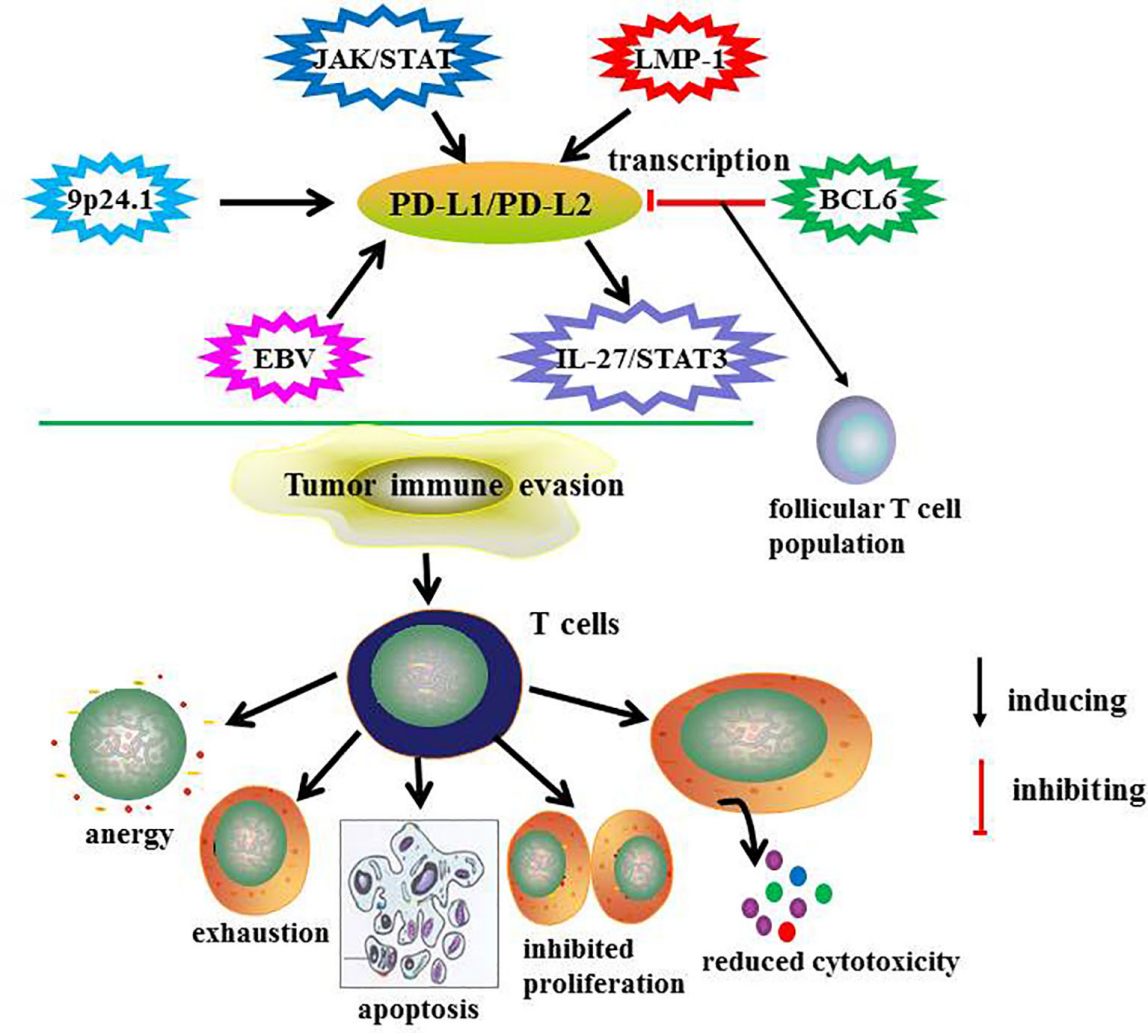
The regulatory mechanisms and function of PD-L1/PD-L2 in lymphoma.

It is reported that PD-L2 was expressed on the surface of malignant cells in 65-100% patients with cHL and 54% of patients with nodular lymphocyte predominant Hodgkin’s lymphoma. Abnormality in chromosome 9p24.1, which encodes PD-L1 and PD-L2 protein and Janus kinase 2, is the main cause of PD-L1 and PD-L2 overexpression (34). Chromosomal rearrangement of PD-L2 is associated with abnormal overexpression in malignant cells of mycosis fungoides (35). BCL6 is a key negative regulator of PD-L1 and PD-L2 in germinal center B cells. It directly binds to the promoter region of PD-L1 and intron 2 of PD-L2 to inhibit its transcription and maintain the size of follicular T cells during the development of germinal center (36). The IL-27/STAT3 signaling pathway induces PD-L1 and PD-L2 expression in infiltrating macrophages of lymphoma (37). PD-L1 and PD-L2 is highly expressed in Epstein-Barr virus (EBV)-positive lymphomas, including DLBCL, extranodal NK/T-cell lymphoma, aggressive NK cell leukemia and T-cell lymphoproliferative diseases (38). Latent membrane protein-1 (LMP1) induced the expression of PD-L1 and PD-L2. Cristino et al. reported that when LMP1 was activated, PD-L1 and PD-L2 expression was significantly increased during the transformation of B cells from the late germinal center to early and late activated B cells. Moreover, microRNA-BHRF1-2-5p plays a regulatory role in LMP1 driven PD-L1 and PD-L2 amplification (39). So further identification of microRNAs that target immune checkpoints allow RNA-based therapy. The regulatory mechanisms of PD-L1 and PD-L2 expression and their function are summarized in **Figure 2**.

cHL prevents immune damage by regulating the interaction between PD-1 and PD-L2 (40). Genetic changes in PD-L2 are rare in non-Hodgkin lymphoma (NHL), in which the expression of PD-L2 protein in non- malignant cells in the tumor microenvironment is higher than that in tumor cells (41). Next-generation sequencing and multivariate analysis has shown that the differential expression of PD-1 and PD-L2 genes in Th-1/Th-2 status guarantees the prognosis of primary central nervous system lymphoma (42). PD-L2 RNA *in situ* hybridization was a sensitive, specific and practical marker to identify primary mediastinal large B-cell lymphoma (PMBCL) (43).

The expression of PD-L2 is correlated with favorable prognosis in patients with DLBCL. Moreover, the high expression levels of PD-L2 are related to low expression of PD-1 and upregulation of CD80 in CD4/CD8 T cells. In addition, fluorescent *in situ* hybridization has shown that changes in the PD-L2 gene were related to the survival rate and gene expression profile of patients (44). However, other studies have reported that PD-L2 expression is associated with poor disease-free survival and overall survival in DLBCL (45). DLBCL with Janus kinases (JAK) 2/PD-L2 amplification shows PMBCL like replication number changes and poor prognosis (46). PD-L2 replication or amplification has been found in the malignant B cells of 64% of patients with T-cell/histiocyte rich large B-cell lymphoma (47). High expression levels of PD-L2 are associated with poor prognosis in FL, while low expression levels are positively correlated with 24-month disease-free survival (48). PD-L2 must be further explored in the future, especially in clinical trials.

## 4 B7-H2

B7-H2 binds to inducible T-cell costimulator (ICOS) and augments Th1 and Th2 function by inducing effector cytokine secretion (49). Few studies have evaluated B7-H2 in hematological tumors, although one found that it is highly expressed in FL B cells that it induced the generation of ICOS+ regulatory T cells, inhibiting the function of conventional T cells (50). In one murine lymphoma model, miR21 is a serum oncogenic biomarker. miR21 indicated that the sensitivity of B cell lymphoma sensitivity to ABT-199 through the ICOS and ICOS ligand signal involved interaction between Treg cells and endothelial cells (51).

## 5 B7-H3

B7-H3 is extensively expressed in various tumors, tumor-infiltrating dendritic cells, and macrophages (52). The exact receptor of B7-H3 remains unclear. Previous studies have reported that myeloid cell-like transcript 2 (TLT-2) binds to B7-H3. However, others found that B7-H3 and TLT2 did not bind to each other (53, 54). A circulating soluble isoform of B7-H3 also exists in serum and other body fluids (55).

Most studies have demonstrated that B7-H3 inhibited T cell function and promoted tumor progression, and one reported that B7-H3 is overexpressed in patients with mantle cell lymphoma (MCL) and cell lines, and that miR-506 negatively regulates the expression of B7-H3, inhibiting cell growth, invasion and migration in MCL. These effects were reversed by the restoration of B7-H3 expression (56). B7-H3 silencing by RNAi suppressed tumor progression and augmented chemosensitivity to chemotherapeutic drugs in U937 cells and MCL cells (57, 58). B7-H3 was correlated with progression-free survival and overall survival time of patients with MM (59, 60). LncRNA NEAT1 sponged miR-214 to induce M2 macrophage polarization by regulating B7-H3, and promoted MM progression through JAK2/STAT3 signaling pathway (59). B7-H3 promoted MM cell survival and growth *via* ROS/Src/c-Cbl signaling pathway (60).

**Figure 3:**
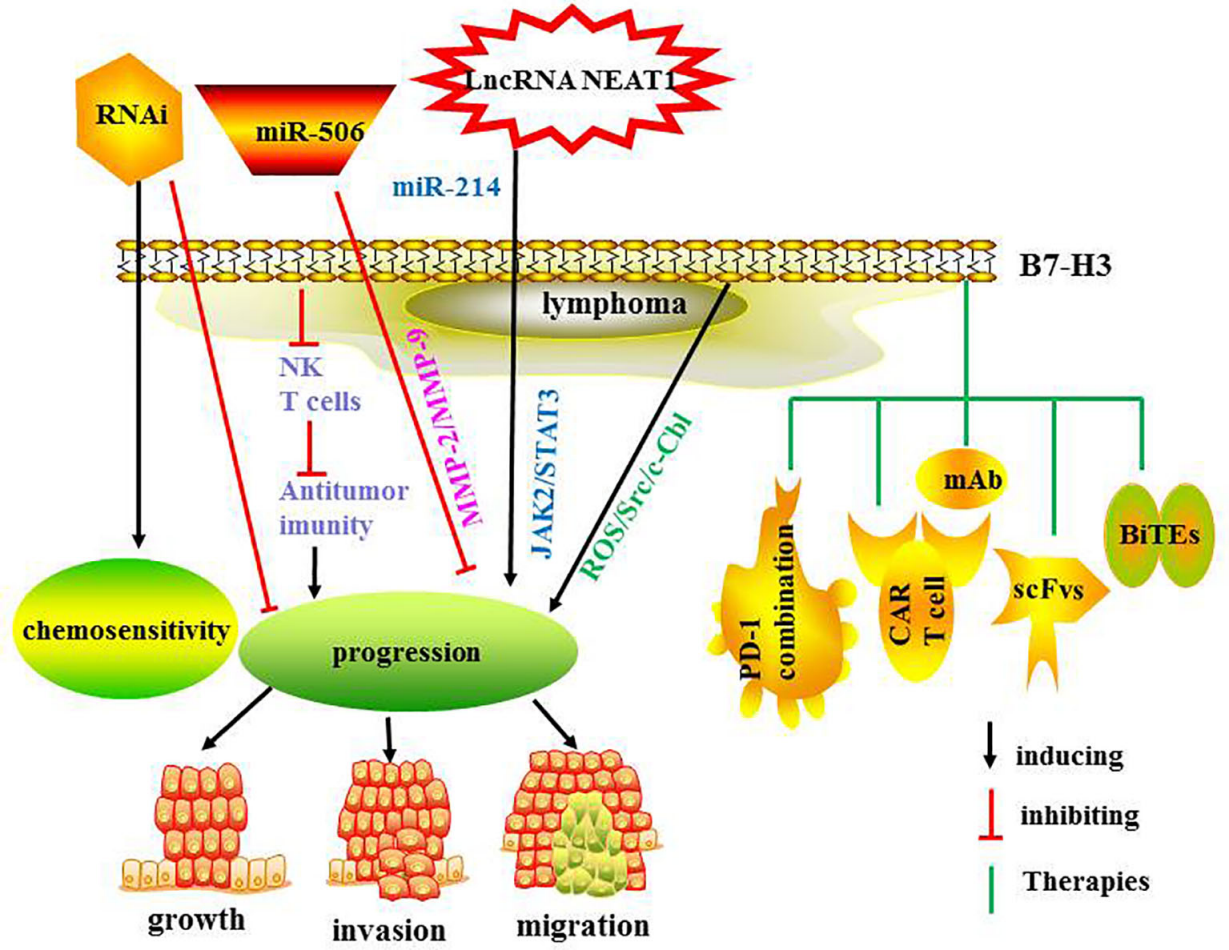
The regulatory mechanisms and therapies targeting B7-H3.

The B7-H3 checkpoint may serve as a promising and novel target for immunotherapy against tumors. B7-H3 inhibition resulted in reduced growth of multiple tumors and enhanced antitumor immunity *via* NK and CD8+ T cells (52). Combining blockades of B7-H3 and PD-1 led to further augmented therapeutic effects on late-stage tumors (52). B7-H3-targeted CAR-T cells showed significant antitumor activity against hematologic malignancies and solid tumors (61). B7-H3 was highly and homogeneously expressed in extranodal nasal NK/T cell lymphoma cell lines. A new anti-B7-H3/CD3 BiTE antibody and B7-H3-redirected CAR-T cells have been constructed. They effectively target and kill NKTCL cells and inhibited the growth of tumors (62). A B7-H3-redirected CAR based on scFvs from mAb 376.96 demonstrated strong cytotoxicity and cytokine production against target anaplastic large cell lymphoma cells *in vitro* and promptly eradicated tumor cells in mouse xenografts. In addition, B7-H3 CAR-T cells show growth capacity and a memory phenotype after stimulation using repeated antigen (63). A bispecific antibody targeting B7-H3 and 4-1BB (B7-H3×4-1BB) has been developed. B7-H3×4-1BB showed antitumor activity in mice and promoted CD8 T cell proliferation and cytokine secretion. B7-H3×4-1BB combined with PD-1 blockade synergistically suppressed tumor growth and increased terminally differentiated CD8 T cells (64). B7-H3 CAR-T cells effectively suppressed tumor growth, both *in vitro* and *in vivo*. B7-H3 CAR and B7-H3/CD16 bispecific killer cell engager (BiKE) have also been generated. B7-H3/CD16 BiKE has been shown to trigger NK cell activity *via* CD16 signaling, enhanced NK cell activation and improved antitumor efficacy *in vitro* and *in vivo* (65). The regulatory mechanisms and therapies targeting B7-H3 are summarized in **Figure 3**. The ongoing clinical trials are summarized in **Table 2**.

Thus, B7-H3 inhibitors may be an effective and safe therapeutic agent against tumors as monotherapy and in combination with other therapeutic agents. Further studies need to be carried out in preclinical and clinical studies.

## 6 B7-H4

B7-H4 negatively modulates T cell immunity and promotes tumor progression (66). The receptor for B7-H4 has not been identified (67). It has been reported that B7-H4 is induced by IL-6, IL-10 and tumor associated macrophages (TAM) and that it protects NHL cells from T cell-mediated killing by secreting IL-6 and IL-10 (68). In another study, B7-H4 augmented the differentiation of mouse leukemia-initiating cells by deleting the phosphatase and tensin homolog in the Akt/RCOR2/RUNX1 signaling pathway (69). In cancer cells, B7-H4 is upregulated by hypoxia *via* hypoxia-inducible factor-1α and promotes tumor cell growth (70).

**Figure 4:**
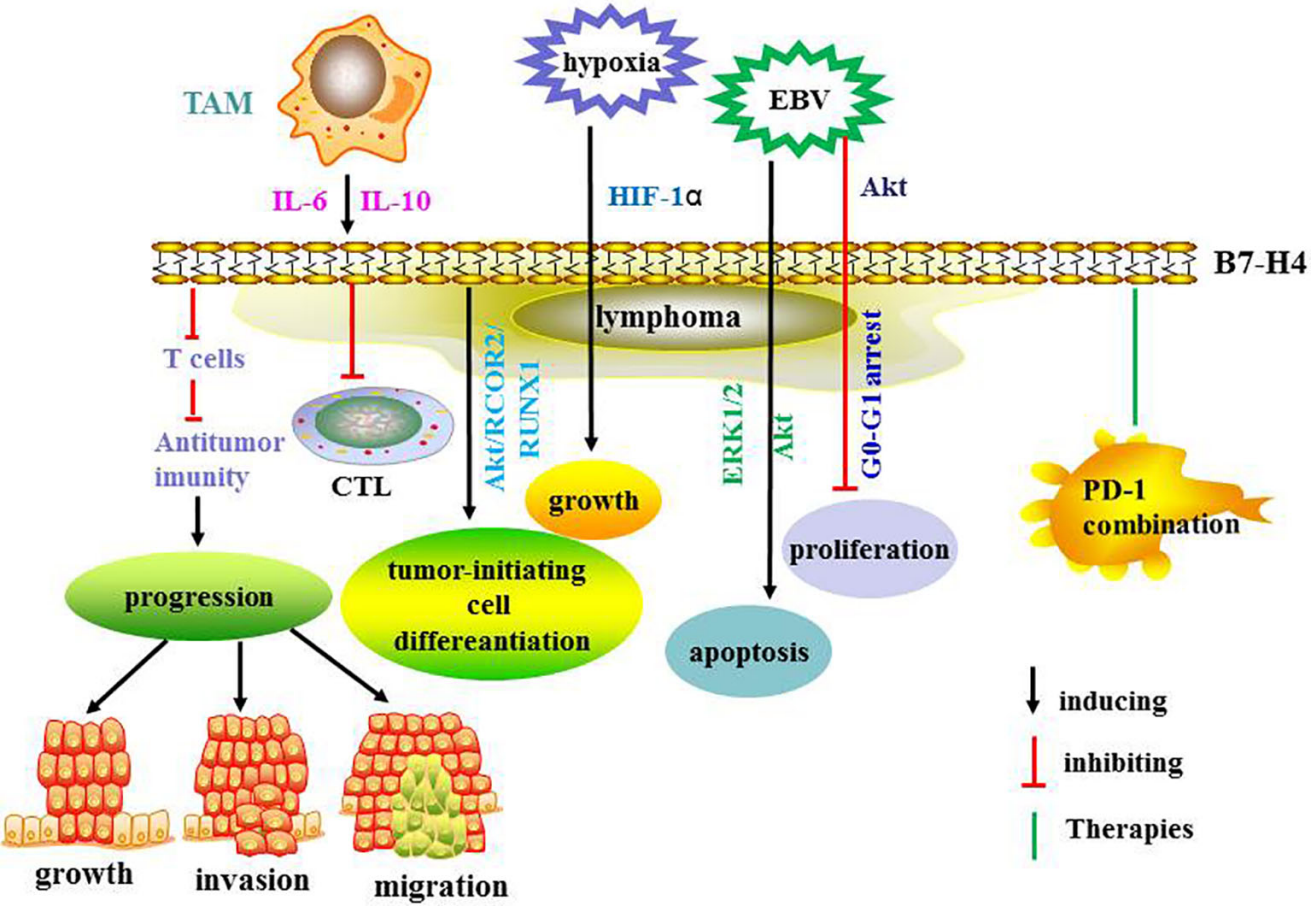
The regulatory mechanisms and therapies targeting B7-H4.

It has been demonstrated that B7-H4 is overexpressed in EBV-positive DLBCL and that it inhibits apoptosis *via* ERK1/2 and Akt signaling pathways (71). Moreover, B7-H4 appears to play a critical role in prognosis while PD-L1 expression weakened (72). One investigation found that B7-H4 engagement in EBV-positive lymphomas inhibited tumor cell proliferation and regulated cell cycle arrest at the G0-G1 phase *via* down-regulation of the Akt signaling pathway (73). Thus, B7-H4 presents as a new potential target for EBV-positive lymphoma immunotherapy. In another study, B7-H4 overexpression in myeloid cells from human cancers was related to CD8+ T cell dysfunction (74). The combination of B7-H4 and PD-1 blockade demonstrated synergic effects and enhanced anti-tumor immune responses (74). Therefore, targeting the B7-H4 co-inhibitory pathway may augment the therapeutic effect of current anti-PD-1 therapy to treat cancers. Recently, it was reported that inhibition of B7-H4 glycosylation recovered antitumor immunity in immune-cold breast cancers (75). Combined with other therapies, this provides a potential insight into new therapeutic strategies. In a study on graft-versus-host disease (GVHD), B7-H4 inhibited T cell function and its expression was increased in GVHD target organs and donor T cells early after bone marrow transplantation (76). The same investigation found that rapid mortality in B7-H4^-/-^ recipients was correlated with increased T cell proliferation, activation, cytokine secretion, and homing in GVHD target tissues (76). Further studies are needed to explore the function of B7-H4 in activated donor T cells, which may offer novel insights and lead to new strategies for the modulating of GVHD. The potential of B7-H4 targeted immunotherapy to treat solid tumors is now being investigated in clinical trials but the results have not yet been reported (67). The same therapy should be further explored in the treatment of lymphoma in preclinical and clinical studies. The regulatory mechanisms and therapies targeting B7-H4 are summarized in **Figure 4**. The ongoing clinical trials are summarized in **Table 2**.

## 7 B7-H6

B7-H6, which is selectively expressed on the tumor cell surface, is a ligand for NKp30, which may be a promising target for novel cancer immunotherapy strategies and has been investigated in CAR-T therapy and novel immunoligands (77). NKp30 induces efficient NK cell-mediated antitumor immune responses triggered by B7-H6 (78). In a recent study, incorporation of affinity-matured B7-H6 into NKp30 therapy enhanced NK cell-mediated tumor cell killing and significantly increased proinflammatory cytokine release of bispecific immunoligands (79).

The expression and regulation of B7-H6 have been explored. In one study, B7-H6 in its soluble and in soluble forms, was induced at the surface of proinflammatory monocytes and neutrophils by ligands of toll-like receptors and proinflammatory cytokines including interleukin-1β and tumor necrosis factor (TNF) α (80). In another investigation, metalloproteases induced B7-H6 release from the tumor cell surface and treatment with metalloprotease inhibitors resulted in both increased surface levels of B7-H6 and augmented NK cell-mediated tumor cell lysis (81). Promoter analyses demonstrated that the proto-oncogene Myc induced B7-H6 expression in tumor cells (78). In one study, suppression of c-Myc or N-Myc markedly reduced the expression levels of B7-H6, and both mRNA and surface protein expression of B7-H6 was reduced by histone deacetylase (HDAC) inhibitors and small interfering RNA-mediated knockdown of HDAC 2 or 3 (82). In another study, B7-H6 downregulation was related to reduced B7-H6 reporter activity and histone acetylation at the B7-H6 promoter (82). Treatment with cisplatin and 5-fluorouracil chemotherapy, radiotherapy, non-lethal heat shock, and TNF-α therapy- induced B7-H6 expression in tumors and enhanced tumor sensitivity to NK cell cytotoxicity (83).

In primary lymphoma tissues, B7-H6 mRNA levels are increased and related to HDAC3 expression (82). HDAC inhibitors reduces B7-H6 expression and NKp30-dependent efficient functions of NK cells. The mRNA levels of c-Myc are significantly correlated with B7-H6 expression, and inhibition of c-Myc damaged NKp30-mediated degranulation of NK cells (78). In B cell NHL, B7-H6 knockdown suppressed tumor progression and enhanced chemosensitivity. Downstream target investigation has indicated that STAT3 pathway is involved in B7-H6 knockdown-mediated antitumor immunity (84). B7-H6 is overexpressed in DLBCL, T-lymphoblastic lymphoma and lymph node reactive hyperplasia tissues promoting cell growth, migration,and invasion through the Ras/MEK/ERK signaling pathway (85).

**Figure 5:**
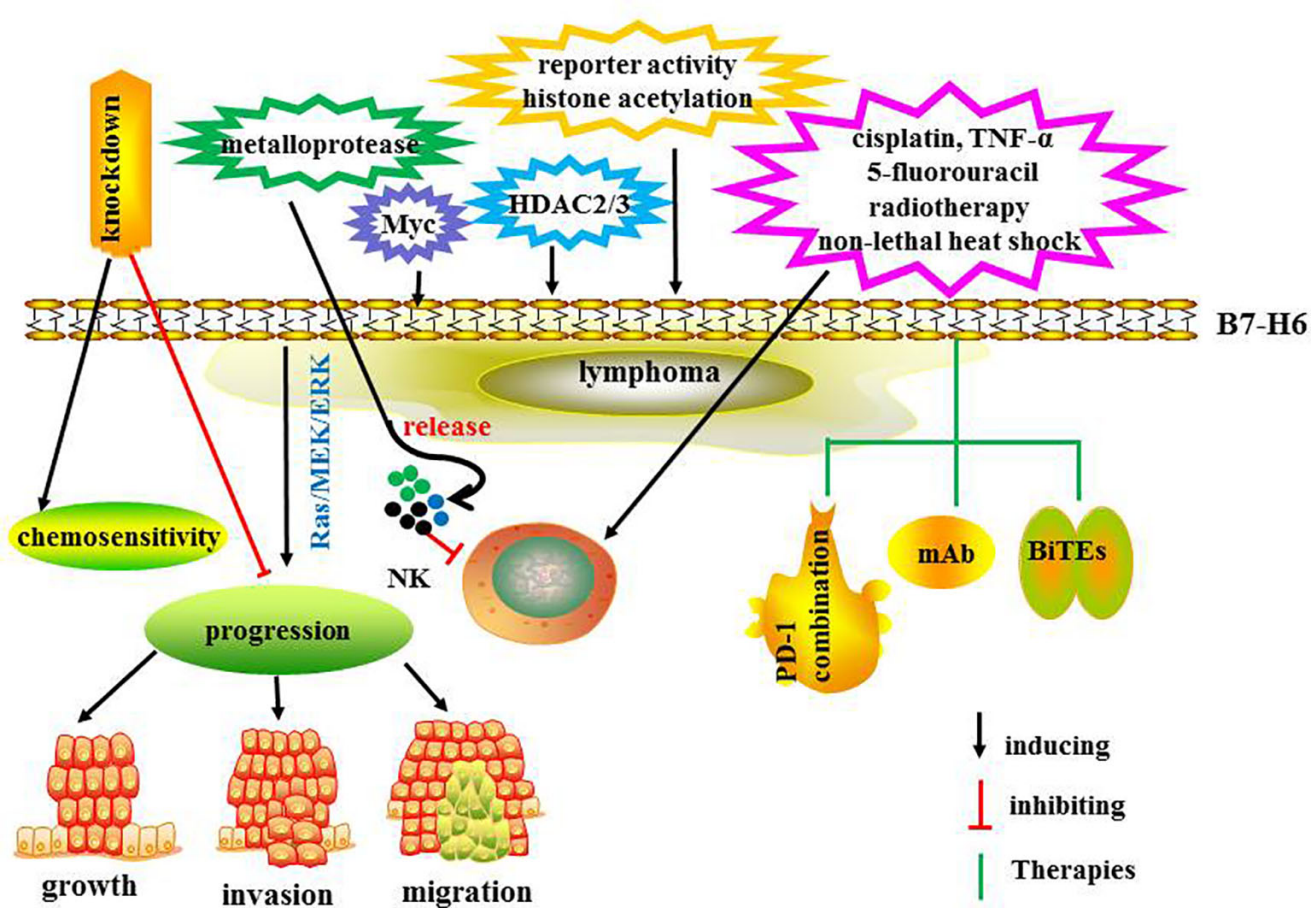
The regulatory mechanisms and therapies targeting B7-H6.

The combination of recombinant immunoligands ULBP2:7D8 and B7-H6:7D8 increases NK cell-mediated ADCC in lymphoma (86). Moreover, bispecific antibody anti-CD3 and anti-B7-H6 (B7-H6Bi-Ab) armed T cells showed significant cytotoxicity induction in B7-H6 positive hematological tumor cells *via* the production of granzyme B and perforin (1). In addition, B7-H6Bi-Ab armed T cells secreted more T cell-derived cytokines and expressed much higher level of the activation marker CD69 (1). Another study demonstrated that B7H6-specific BiTEs directed T cells to mediate cytolysis and IFN-γ production against tumors (87). *In vivo*, B7-H6-specific BiTE significantly increases the survival of lymphoma-bearing mice *via* perforin and IFN-γ secretion. Moreover, BiTE protein reduces tumor burden in melanoma and ovarian cancer-bearing mice. Therefore, combining therapeutic antibodies may provide a promising insight to further enhance the efficacy of antibody therapy. This strategy may be especially encouraging for eradicating minimal residual disease cells after transplantation. The regulatory mechanisms and therapies targeting B7-H36 are summarized in **Figure 5**. The ongoing clinical trials are summarized in **Table 2**. Taken together, B7-H6 may be a promising immunotherapy target for hematological and solid tumors. Further explorations of B7-H6 targeted immunotherapy should be carried out in the preclinical and clinical studies.

## Conclusion

In summary, B7 family members may provide novel strategies to inhibit or kill tumors by triggering antitumor immune responses. Blocking the PD-L1/PD-1 pathway has generated therapeutic success in human tumors. However, PD-L1/PD-1 have a low response rate. Therefore, researchers must combine novel checkpoint inhibitors with PD-L1/PD-1 inhibitors, or used them in monotherapy. The B7 family member pathways represent novel immunosuppressive mechanisms in tumor immunity, as well as a potential target for immunotherapy. Additional studies, involving immunoregulatory mechanisms and clinical trials are needed for further exploration. In the future, immunotherapy based on combined B7 family members may represent a promising strategy for treating hematologic malignancies.

## Author Contributions

WZ and YQ designed the study and wrote the manuscript. YF and XX participated in data acquisition and statistics analysis. LW and ZC edited and revised the manuscript. All authors contributed to the article and approved the submitted version.

## Funding

This work was supported by grants from the Key Research Project Program of Shandong Province [2018GSF118035], the Chinese Medical Health Science and Technology Development Plan of Shandong Province [2017-462], the Medical Health Science and Technology Development Plan of Shandong Province [2017WS638], Linyi Science and Technology Development Project [201919019] and Development Fund of Affiliated Hospital of Xuzhou Medical University [XYFM2020016].

## Conflict of Interest

The authors declare that the research was conducted in the absence of any commercial or financial relationships that could be construed as a potential conflict of interest.

## Abbreviations

ADC, antibody-drug conjugate; B7-H3×4-1BB bispecific antibody targeting on B7-H3 and 4-1BB; B7-H6Bi-Ab, bispecific antibody anti-CD3 and anti-B7-H6; BiKE, bispecific killer cell engager; BiTEs, bispecific T cell engagers; CAR, chimeric antigen receptor; cHL, classic Hodgkin lymphoma; CR, complete response; CTLA-4, cytotoxic T lymphocyte antigen 4; DLBCL, diffuse large B cell lymphoma; EBV, Epstein-Barr virus; ENKTL, extranodal NK/T cell lymphoma; FL, follicular lymphoma; GVHD, graft-versus-host disease; HDAC, histone deacetylase; ICOS, inducible T-cell costimulator; JAK, Janus Kinases; LMP1, Latent membrane protein-1; mAb, monoclonal antibody; MCL, mantle cell lymphoma; MM, multiple myeloma; MRD, minimal residual disease; NHL, non Hodgkin lymphoma; NK, natural killer; ORR, overall response rate; PD-1, programmed cell death protein-1; PD-L1, programmed death ligand-1; PD-L2, programmed death ligand-2; PMBCL, primary mediastinal large B-cell lymphoma; scFvs, single chain fragment variables; SCT, stem cell transplantation; STAT, signal transducer and activator of transcription; TAM, tumor associated macrophages; TLT-2, myeloid cell like transcript 2; TNF, tumor necrosis factor.

## References

[1] Sun X Zhao J Ma L Sun X Ge J Yu Y. B7-H6 as an efficient target for T cell-induced cytotoxicity in haematologic malignant cells. Invest New Drugs (2020) 39(1):24–33. 10.1007/s10637-020-00976-5 32770284

[2] Andrews LP Yano H Vignali DAA. Inhibitory receptors and ligands beyond PD-1, PD-L1 and CTLA-4: breakthroughs or backups. Nat Immunol (2019) 20(11):1425–34. 10.1038/s41590-019-0512-0 31611702

[3] Wang C Feng H Cheng X Liu K Cai D Zhao R. Potential Therapeutic Targets of B7 Family in Colorectal Cancer. Front Immunol (2020) 11:681. 10.3389/fimmu.2020.00681 32477326 PMC7232583

[4] Kaur G Janakiram M. B7x-from bench to bedside. ESMO Open (2019) 4(5):e000554. 10.1136/esmoopen-2019-000554 31555486 PMC6735664

[5] Zhang L Mai W Jiang W Geng Q. Sintilimab: A Promising Anti-Tumor PD-1 Antibody. Front Oncol (2020) 10:594558. 10.3389/fonc.2020.594558 33324564 PMC7726413

[6] Xue Y Gao S Gou J Yin T He H Wang Y. Platinum-based chemotherapy in combination with PD-1/PD-L1 inhibitors: preclinical and clinical studies and mechanism of action. Expert Opin Drug Delivery (2020) 18(2):1–17. 10.1080/17425247.2021.1825376 32954856

[7] Lin N Song Y Zhu J. Immune checkpoint inhibitors in malignant lymphoma: Advances and perspectives. Chin J Cancer Res (2020) 32(3):303–18. 10.21147/j.issn.1000-9604.2020.03.03 PMC7369179 32694896

[8] Shi Y Su H Song Y Jiang W Sun X Qian W. Safety and activity of sintilimab in patients with relapsed or refractory classical Hodgkin lymphoma (ORIENT-1): a multicentre, single-arm, phase 2 trial. Lancet Haematol (2019) 6(1):e12–9. 10.1016/S2352-3026(18)30192-3 30612710

[9] Zheng R Chen X Wang C Qin P Tan H Luo X. Triplet Therapy with PD-1 Blockade, Histone Deacetylase Inhibitor, and DNA Methyltransferase Inhibitor Achieves Radiological Response in Refractory Double-Expressor Diffuse Large B-cell Lymphoma with 17p Deletion. Case Rep Hematol (2020) 2020:8879448. 10.1155/2020/8879448 32908729 PMC7471814

[10] Younes A Santoro A Shipp M Zinzani PL Timmerman JM Ansell S. Nivolumab for classical Hodgkin’s lymphoma after failure of both autologous stem-cell transplantation and brentuximab vedotin: a multicentre, multicohort, single-arm phase 2 trial. Lancet Oncol (2016) 17(9):1283–94. 10.1016/S1470-2045(16)30167-X PMC5541855 27451390

[11] Chen R Zinzani PL Fanale MA Armand P Johnson NA Brice P. Phase II Study of the Efficacy and Safety of Pembrolizumab for Relapsed/Refractory Classic Hodgkin Lymphoma. J Clin Oncol (2017) 35(19):2125–32. 10.1200/JCO.2016.72.1316 PMC5791843 28441111

[12] Li X Cheng Y Zhang M Yan J Li L Fu X. Activity of pembrolizumab in relapsed/refractory NK/T-cell lymphoma. J Hematol Oncol (2018) 11(1):15. 10.1186/s13045-018-0559-7 29386072 PMC5793390

[13] Shi Y Wu J Wang Z Zhang L Wang Z Zhang M. Efficacy and safety of geptanolimab (GB226) for relapsed or refractory peripheral T cell lymphoma: an open-label phase 2 study (Gxplore-002). J Hematol Oncol (2021) 14(1):12. 10.1186/s13045-021-01033-1 33436023 PMC7802130

[14] Kim SJ Lim JQ Laurensia Y Cho J Yoon SE Lee JY. Avelumab for the treatment of relapsed or refractory extranodal NK/T-cell lymphoma: an open-label phase 2 study. Blood (2020) 136(24):2754–63. 10.1182/blood.2020007247 32766875

[15] Herrera AF Goy A Mehta A Ramchandren R Pagel JM Svoboda J. Safety and activity of ibrutinib in combination with durvalumab in patients with relapsed or refractory follicular lymphoma or diffuse large B-cell lymphoma. Am J Hematol (2020) 95(1):18–27. 10.1002/ajh.25659 31621094 PMC6904508

[16] Geoerger B. Atezolizumab for children and young adults with previously treated solid tumours, non-Hodgkin lymphoma, and Hodgkin lymphoma (iMATRIX): a multicentre phase 1-2 study. Lancet Oncol (2020) 21(1):134–44. 10.1016/S1470-2045(19)30693-X 31780255

[17] Liu H Lei W Zhang C Yang C Wei J Guo Q. CD19-specific CAR T Cells that Express a PD-1/CD28 Chimeric Switch-Receptor are Effective in Patients with PD-L1-positive B-Cell Lymphoma. Clin Cancer Res (2021) 27(2):473–84. 10.1158/1078-0432.CCR-20-1457 33028589

[18] Ansell SM Lesokhin AM Borrello I Halwani A Scott EC Gutierrez M. PD-1 blockade with nivolumab in relapsed or refractory Hodgkin’s lymphoma. N Engl J Med (2015) 372(4):311–9. 10.1056/NEJMoa1411087 PMC4348009 25482239

[19] Younes A Brody J Carpio C Lopez-Guillermo A Ben-Yehuda D Ferhanoglu B. Safety and activity of ibrutinib in combination with nivolumab in patients with relapsed non-Hodgkin lymphoma or chronic lymphocytic leukaemia: a phase 1/2a study. Lancet Haematol (2019) 6(2):e67–78. 10.1016/S2352-3026(18)30217-5 30642819

[20] Armand P Shipp MA Ribrag V Michot JM Zinzani PL Kuruvilla J. Programmed Death-1 Blockade With Pembrolizumab in Patients With Classical Hodgkin Lymphoma After Brentuximab Vedotin Failure. J Clin Oncol (2016) 34(31):3733–9. 10.1200/JCO.2016.67.3467 PMC5791838 27354476

[21] Armand P Rodig S Melnichenko V Thieblemont C Bouabdallah K Tumyan G. Pembrolizumab in Relapsed or Refractory Primary Mediastinal Large B-Cell Lymphoma. J Clin Oncol (2019) 37(34):3291–9. 10.1200/JCO.19.01389 PMC6881098 31609651

[22] Ding W LaPlant BR Call TG Parikh SA Leis JF He R. Pembrolizumab in patients with CLL and Richter transformation or with relapsed CLL. Blood (2017) 129(26):3419–27. 10.1182/blood-2017-02-765685 PMC5492091 28424162

[23] Chen R Zinzani PL Lee HJ Armand P Johnson NA Brice P. Pembrolizumab in relapsed or refractory Hodgkin lymphoma: 2-year follow-up of KEYNOTE-087. Blood (2019) 134(14):1144–53. 10.1182/blood.2019000324 PMC6776792 31409671

[24] Smith SD Till BG Shadman MS Lynch RC Cowan AJ Wu QV. Pembrolizumab with R-CHOP in previously untreated diffuse large B-cell lymphoma: potential for biomarker driven therapy. Br J Haematol (2020) 189(6):1119–26. 10.1111/bjh.16494 32030732

[25] Liu Y Barta SK. Diffuse large B-cell lymphoma: 2019 update on diagnosis, risk stratification, and treatment. Am J Hematol (2019) 94(5):604–16. 10.1002/ajh.25460 30859597

[26] Song Y Wu J Chen X Lin T Cao J Liu Y. Single-Arm A. Multicenter, Phase II Study of Camrelizumab in Relapsed or Refractory Classical Hodgkin Lymphoma. Clin Cancer Res (2019) 25(24):7363–9. 10.1158/1078-0432.CCR-19-1680 31420358

[27] Chen J Zhang H Zhu L Zhao Y Ding Y Yuan Y. Tislelizumab for the treatment of classical Hodgkin’s lymphoma. Drugs Today (Barc) (2020) 56(12):781–5. 10.1358/dot.2020.56.12.3233362 33332484

[28] Zinzani PL Santoro A Gritti G Brice P Barr PM Kuruvilla J. Nivolumab Combined With Brentuximab Vedotin for Relapsed/Refractory Primary Mediastinal Large B-Cell Lymphoma: Efficacy and Safety From the Phase II CheckMate 436 Study. J Clin Oncol (2019) 37(33):3081–9. 10.1200/JCO.19.01492 PMC6864847 31398081

[29] Davids MS Kim HT Bachireddy P Costello C Liguori R Savell A. Ipilimumab for Patients with Relapse after Allogeneic Transplantation. N Engl J Med (2016) 375(2):143–53. 10.1056/NEJMoa1601202 PMC5149459 27410923

[30] Fei Y Yu J Li Y Li L Zhou S Zhang T. Plasma soluble PD-L1 and STAT3 predict the prognosis in diffuse large B cell lymphoma patients. J Cancer (2020) 11(23):7001–8. 10.7150/jca.47816 PMC7591999 33123290

[31] Wei T Li M Zhu Z Xiong H Shen H Zhang H. Vincristine upregulates PD-L1 and increases the efficacy of PD-L1 blockade therapy in diffuse large B-cell lymphoma. J Cancer Res Clin Oncol (2021) 147(3):691–701. 10.1007/s00432-020-03446-w 33389078 PMC11801829

[32] Zhang XW Bi XW Liu PP Liu ZL Nie M Yang H. Expression of PD-L1 on Monocytes Is a Novel Predictor of Prognosis in Natural Killer/T-Cell Lymphoma. Front Oncol (2020) 10:1360. 10.3389/fonc.2020.01360 32850435 PMC7424071

[33] Korkmaz S Erdem S Akay E Taşdemir EA Karaman H Keklik M. Do PD-1 and PD-L2 expressions have prognostic impact in hematologic malignancies? Turk J Med Sci (2019) 49(1):265–71. 10.3906/sag-1706-194 PMC7350792 30761875

[34] Jelinek T Mihalyova J Kascak M Duras J Hajek R. PD-1/PD-L1 inhibitors in haematological malignancies: update 2017. Immunology (2017) 152(3):357–71. 10.1111/imm.12788 PMC5629439 28685821

[35] Lesokhin AM Ansell SM Armand P Scott EC Halwani A Gutierrez M. Nivolumab in Patients With Relapsed or Refractory Hematologic Malignancy: Preliminary Results of a Phase Ib Study. J Clin Oncol (2016) 34(23):2698–704. 10.1200/JCO.2015.65.9789 PMC5019749 27269947

[36] Peng C Hu Q Yang F Zhang H Li F Huang C. BCL6-Mediated Silencing of PD-1 Ligands in Germinal Center B Cells Maintains Follicular T Cell Population. J Immunol (2019) 202(3):704–13. 10.4049/jimmunol.1800876 30567732

[37] Horlad H Ma C Yano H Pan C Ohnishi K Fujiwara Y. An IL-27/Stat3 axis induces expression of programmed cell death 1 ligands (PD-L1/2) on infiltrating macrophages in lymphoma. Cancer Sci (2016) 107(11):1696–704. 10.1111/cas.13065 PMC5132271 27564404

[38] Kataoka K Miyoshi H Sakata S Dobashi A Couronné L Kogure Y. Frequent structural variations involving programmed death ligands in Epstein-Barr virus-associated lymphomas. Leukemia (2019) 33(7):1687–99. 10.1038/s41375-019-0380-5 PMC6755969 30683910

[39] Cristino AS Nourse J West RA Sabdia MB Law SC Gunawardana J. EBV microRNA-BHRF1-2-5p targets the 3’UTR of immune checkpoint ligands PD-L1 and PD-L2. Blood (2019) 134(25):2261–70. 10.1182/blood.2019000889 PMC6923667 31856276

[40] Al Hadidi,SA Lee HJ. Pembrolizumab for the treatment of Hodgkin Lymphoma. Expert Opin Biol Ther (2020) 20(11):1275–82. 10.1080/14712598.2020.1830056 33006479

[41] Furuse M Kuwabara H Ikeda N Hattori Y Ichikawa T Kagawa N. PD-L1 and PD-L2 expression in the tumor microenvironment including peritumoral tissue in primary central nervous system lymphoma. BMC Cancer (2020) 20(1):277. 10.1186/s12885-020-06755-y 32248797 PMC7132991

[42] Takashima Y Kawaguchi A Sato R Yoshida K Hayano A Homma J. Differential expression of individual transcript variants of PD-1 and PD-L2 genes on Th-1/Th-2 status is guaranteed for prognosis prediction in PCNSL. Sci Rep (2019) 9(1):10004. 10.1038/s41598-019-46473-5 31292525 PMC6620277

[43] Wang Z Cook JR. PDCD1LG2 (PD-L2) RNA *in situ* hybridization is a sensitive, specific, and practical marker of primary mediastinal large B-cell lymphoma. Br J Haematol (2018) 181(4):564–6. 10.1111/bjh.14670 28369778

[44] Xu-Monette ZY Xiao M Au Q Padmanabhan R Xu B Hoe N. Immune Profiling and Quantitative Analysis Decipher the Clinical Role of Immune-Checkpoint Expression in the Tumor Immune Microenvironment of DLBCL. Cancer Immunol Res (2019) 7(4):644–57. 10.1158/2326-6066.CIR-18-0439 30745366

[45] Krittikarux S Wudhikarn K Tangnuntachai N Assanasen T Sukswai N Asawapanumas T. The influence of programmed cell death ligand 2 (PD-L2) expression on survival outcome and tumor microenvironment in diffuse large B cell lymphoma. Leuk Lymphoma (2020) p:1–9. 10.1080/10428194.2020.1808209 32820659

[46] Xue X Huang W Qiu T Guo L Ying J Lv N. DLBCL with amplification of JAK2/PD-L2 exhibits PMBCL-like CNA pattern and worse clinical outcome resembling those with MYD88 L265P mutation. BMC Cancer (2020) 20(1):816. 10.1186/s12885-020-07293-3 32854650 PMC7450805

[47] Griffin GK Weirather JL Roemer MGM Lipschitz M Kelley A Chen PH. Spatial Signatures Identify Immune Escape *via* PD-1 as a Defining Feature of T-cell/Histiocyte-rich Large B-cell Lymphoma. Blood (2020) 137(10):1353–64. 10.1182/blood.2020006464 PMC8555417 32871584

[48] Tobin JWD Keane C Gunawardana J Mollee P Birch S Hoang T. Progression of Disease Within 24 Months in Follicular Lymphoma Is Associated With Reduced Intratumoral Immune Infiltration. J Clin Oncol (2019) 37(34):3300–9. 10.1200/JCO.18.02365 PMC6881104 31461379

[49] Burugu S Dancsok AR Nielsen TO. Emerging targets in cancer immunotherapy. Semin Cancer Biol (2018) 52(Pt 2):39–52. 10.1016/j.semcancer.2017.10.001 28987965

[50] Le K-S Thibult ML Just-Landi S Pastor S Gondois-Rey F Granjeaud S. Follicular B Lymphomas Generate Regulatory T Cells *via* the ICOS/ICOSL Pathway and Are Susceptible to Treatment by Anti-ICOS/ICOSL Therapy. Cancer Res (2016) 76(16):4648–60. 10.1158/0008-5472.CAN-15-0589 27246829

[51] Zheng Z Xu PP Wang L Zhao HJ Weng ZQ Zhong HJ. MiR21 sensitized B-lymphoma cells to ABT-199 *via* ICOS/ICOSL-mediated interaction of Treg cells with endothelial cells. J Exp Clin Cancer Res: CR (2017) 36(1):82. 10.1186/s13046-017-0551-z 28637496 PMC5480196

[52] Lee YH Martin-Orozco N Zheng P Li J Zhang P Tan H. Inhibition of the B7-H3 immune checkpoint limits tumor growth by enhancing cytotoxic lymphocyte function. Cell Res (2017) 27(8):1034–45. 10.1038/cr.2017.90 PMC5539354 28685773

[53] Li G Quan Y Che F Wang L. B7-H3 in tumors: friend or foe for tumor immunity? Cancer Chemother Pharmacol (2018) 81(2):245–53. 10.1007/s00280-017-3508-1 29299639

[54] Michelakos T Kontos F Barakat O Maggs L Schwab JH Ferrone CR. B7-H3 targeted antibody-based immunotherapy of malignant diseases. Expert Opin Biol Ther (2020) p:1–16. 10.1080/14712598.2021.1862791 PMC8087627 33301369

[55] Flem-Karlsen K Fodstad O Nunes-Xavier CE. B7-H3 Immune Checkpoint Protein in Human Cancer. Curr Med Chem (2020) 27(24):4062–86. 10.2174/0929867326666190517115515 31099317

[56] Zhu XW Wang J Zhu MX Wang YF Yang SY Ke XY. MicroRNA-506 inhibits the proliferation and invasion of mantle cell lymphoma cells by targeting B7H3. Biochem Biophys Res Commun (2019) 508(4):1067–73. 10.1016/j.bbrc.2018.12.055 30553455

[57] Zhang W Wang J Wang Y Dong F Zhu M Wan W. B7-H3 silencing by RNAi inhibits tumor progression and enhances chemosensitivity in U937 cells. Onco Targets Ther (2015) 8:1721–33. 10.2147/OTT.S85272 PMC4508088 26203263

[58] Zhang W Wang Y Wang J Dong F Zhu M Wan W. B7-H3 silencing inhibits tumor progression of mantle cell lymphoma and enhances chemosensitivity. Int J Oncol (2015) 46(6):2562–72. 10.3892/ijo.2015.2962 25872657

[59] Gao Y Fang P Li WJ Zhang J Wang GP Jiang DF. LncRNA NEAT1 sponges miR-214 to regulate M2 macrophage polarization by regulation of B7-H3 in multiple myeloma. Mol Immunol (2020) 117:20–8. 10.1016/j.molimm.2019.10.026 31731055

[60] Lin L Cao L Liu Y Wang K Zhang X Qin X. B7-H3 promotes multiple myeloma cell survival and proliferation by ROS-dependent activation of Src/STAT3 and c-Cbl-mediated degradation of SOCS3. Leukemia (2019) 33(6):1475–86. 10.1038/s41375-018-0331-6 30573782

[61] Zhang Z Jiang C Liu Z Yang M Tang X Wang Y. B7-H3-Targeted CAR-T Cells Exhibit Potent Antitumor Effects on Hematologic and Solid Tumors. Mol Ther Oncolytics (2020) 17:180–9. 10.1016/j.omto.2020.03.019 PMC7178328 32346608

[62] Zheng M Yu L Hu J Zhang Z Wang H Lu D. Efficacy of B7-H3-Redirected BiTE and CAR-T Immunotherapies Against Extranodal Nasal Natural Killer/T Cell Lymphoma. Transl Oncol (2020) 13(5):100770. 10.1016/j.tranon.2020.100770 32298986 PMC7160598

[63] Zi Z Zhao H Wang H Ma X Wei F. B7-H3 Chimeric Antigen Receptor Redirected T Cells Target Anaplastic Lymphoma Kinase-Positive Anaplastic Large Cell Lymphoma. Cancers (Basel) (2020) 12(12). 10.3390/cancers12123815 PMC7766167 33348781

[64] You G Lee Y Kang YW Park HW Park K Kim H. B7-H3x4-1BB bispecific antibody augments antitumor immunity by enhancing terminally differentiated CD8(+) tumor-infiltrating lymphocytes. Sci Adv (2021) 7(3):eaax3160. 10.1126/sciadv.aax3160 33523913 PMC7810375

[65] Liu J Yang S Cao B Zhou G Zhang F Wang Y. Targeting B7-H3 *via* chimeric antigen receptor T cells and bispecific killer cell engagers augments antitumor response of cytotoxic lymphocytes. J Hematol Oncol (2021) 14(1):21. 10.1186/s13045-020-01024-8 33514401 PMC7844995

[66] Sica GL Choi IH Zhu G Tamada K Wang SD Tamura H. B7-H4, a molecule of the B7 family, negatively regulates T cell immunity. Immunity (2003) 18(6):849–61. 10.1016/S1074-7613(03)00152-3 12818165

[67] John P Wei Y Liu W Du M Guan F Zang X. The B7x Immune Checkpoint Pathway: From Discovery to Clinical Trial. Trends Pharmacol Sci (2019) 40(11):883–96. 10.1016/j.tips.2019.09.008 PMC6907741 31677920

[68] Che F Heng X Zhang H Su Q Zhang B Chen Y. Novel B7-H4-mediated crosstalk between human non-Hodgkin lymphoma cells and tumor-associated macrophages leads to immune evasion *via* secretion of IL-6 and IL-10. Cancer Immunol Immunother (2017) 66(6):717–29. 10.1007/s00262-017-1961-7 PMC11028477 28246881

[69] Xia F Zhang Y Xie L Jiang H Zeng H Chen C. B7-H4 enhances the differentiation of murine leukemia-initiating cells *via* the PTEN/AKT/RCOR2/RUNX1 pathways. Leukemia (2017) 31(10):2260–4. 10.1038/leu.2017.232 PMC5629360 28744012

[70] Jeon YK Park SG Choi IW Lee SW Lee SM Choi I. Cancer cell-associated cytoplasmic B7-H4 is induced by hypoxia through hypoxia-inducible factor-1alpha and promotes cancer cell proliferation. Biochem Biophys Res Commun (2015) 459(2):277–83. 10.1016/j.bbrc.2015.02.098 25725157

[71] Jiang Y Cai G Lin J Zhang J Bo Z Li Y. B7-H4 is highly expressed in aggressive Epstein-Barr virus positive diffuse large B-cell lymphoma and inhibits apoptosis through upregulating Erk1/2 and Akt signalling pathways. Infect Agent Cancer (2019) 14:20. 10.1186/s13027-019-0234-9 31406503 PMC6686556

[72] Jiang Y Lin J Zhang J Lu S Wang C Tong Y. Expression of co-inhibitory molecules B7-H4 and B7-H1 in Epstein-Barr virus positive diffuse large B-cell lymphoma and their roles in tumor invasion. Pathol Res Pract (2019) 215(12):152684. 10.1016/j.prp.2019.152684 31679792

[73] Park GB Song H Kim YS Sung M Ryu JW Lee HK. Cell cycle arrest induced by engagement of B7-H4 on Epstein-Barr virus-positive B-cell lymphoma cell lines. Immunology (2009) 128(3):360–8. 10.1111/j.1365-2567.2009.03111.x PMC2770684 20067536

[74] Li J Lee Y Li Y Jiang Y Lu H Zang W. Co-inhibitory Molecule B7 Superfamily Member 1 Expressed by Tumor-Infiltrating Myeloid Cells Induces Dysfunction of Anti-tumor CD8(+) T Cells. Immunity (2018) 48(4):773–86. 10.1016/j.immuni.2018.03.018 29625896

[75] Song X Zhou Z Li H Xue Y Lu X Bahar I. Pharmacologic Suppression of B7-H4 Glycosylation Restores Antitumor Immunity in Immune-Cold Breast Cancers. Cancer Discovery (2020) 10(12):1872–93. 10.1158/2159-8290.CD-20-0402 PMC7710601 32938586

[76] Saha A Taylor PA Lees CJ Panoskaltsis-Mortari A Osborn MJ Feser CJ. Donor and host B7-H4 expression negatively regulates acute graft-versus-host disease lethality. JCI Insight (2019) 4(19):e127716. 10.1172/jci.insight.127716 PMC6795410 31578305

[77] Pinheiro PF Justino GC Marques MM. NKp30 - A prospective target for new cancer immunotherapy strategies. Br J Pharmacol (2020) 177(20):4563–80. 10.1111/bph.15222 PMC7520444 32737988

[78] Textor S Bossler F Henrich KO Gartlgruber M Pollmann J Fiegler N. The proto-oncogene Myc drives expression of the NK cell-activating NKp30 ligand B7-H6 in tumor cells. Oncoimmunology (2016) 5(7):e1116674. 10.1080/2162402X.2015.1116674 27622013 PMC5007025

[79] Pekar L Klausz K Busch M Valldorf B Kolmar H Wesch D. Affinity Maturation of B7-H6 Translates into Enhanced NK Cell-Mediated Tumor Cell Lysis and Improved Proinflammatory Cytokine Release of Bispecific Immunoligands *via* NKp30 Engagement. J Immunol (2020) 206(1):225–36. 10.4049/jimmunol.2001004 PMC7750860 33268483

[80] Matta J Baratin M Chiche L Forel JM Cognet C Thomas G. Induction of B7-H6, a ligand for the natural killer cell-activating receptor NKp30, in inflammatory conditions. Blood (2013) 122(3):394–404. 10.1182/blood-2013-01-481705 23687088

[81] Schlecker E Fiegler N Arnold A Altevogt P Rose-John S Moldenhauer G. Metalloprotease-mediated tumor cell shedding of B7-H6, the ligand of the natural killer cell-activating receptor NKp30. Cancer Res (2014) 74(13):3429–40. 10.1158/0008-5472.CAN-13-3017 24780758

[82] Fiegler N Textor S Arnold A Rölle A Oehme I Breuhahn K. Downregulation of the activating NKp30 ligand B7-H6 by HDAC inhibitors impairs tumor cell recognition by NK cells. Blood (2013) 122(5):684–93. 10.1182/blood-2013-02-482513 23801635

[83] Cao G Wang J Zheng X Wei H Tian Z Sun R. Tumor Therapeutics Work as Stress Inducers to Enhance Tumor Sensitivity to Natural Killer (NK) Cell Cytolysis by Up-regulating NKp30 Ligand B7-H6. J Biol Chem (2015) 290(50):29964–73. 10.1074/jbc.M115.674010 PMC4705966 26472927

[84] Wu F Wang J Ke X. Knockdown of B7-H6 inhibits tumor progression and enhances chemosensitivity in B-cell non-Hodgkin lymphoma. Int J Oncol (2016) 48(4):1561–70. 10.3892/ijo.2016.3393 26891663

[85] Yang S Yuan L Wang Y Zhu M Wang J Ke X. B7-H6 Promotes Cell Proliferation, Migration and Invasion of Non-Hodgkin Lymphoma *via* Ras/MEK/ERK Pathway Based on Quantitative Phosphoproteomics Data. Onco Targets Ther (2020) 13:5795–805. 10.2147/OTT.S257512 PMC7308182 32606790

[86] Kellner C Günther A Humpe A Repp R Klausz K Derer S. Enhancing natural killer cell-mediated lysis of lymphoma cells by combining therapeutic antibodies with CD20-specific immunoligands engaging NKG2D or NKp30. Oncoimmunology (2016) 5(1):e1058459. 10.1080/2162402X.2015.1058459 26942070 PMC4760288

[87] Wu MR Zhang T Gacerez AT Coupet TA DeMars LR Sentman CL. B7H6-Specific Bispecific T Cell Engagers Lead to Tumor Elimination and Host Antitumor Immunity. J Immunol (2015) 194(11):5305–11. 10.4049/jimmunol.1402517 PMC4433849 25911747

